# Therapeutic Effect of Intrastromal Voriconazole, Topical Voriconazole, and Topical Natamycin on* Fusarium* Keratitis in Rabbit

**DOI:** 10.1155/2016/8692830

**Published:** 2016-05-19

**Authors:** Mahmood Nejabat, Nafiseh Yaqubi, Amir Khosravi, Kamiar Zomorodian, Mohammad Javad Ashraf, Ramin Salouti

**Affiliations:** ^1^Poostchi Eye Research Center, Department of Ophthalmology, Shiraz University of Medical Sciences, Shiraz 7134845794, Iran; ^2^Department of Ophthalmology, Shiraz University of Medical Sciences, Shiraz 7134845794, Iran; ^3^Yasuj University of Medical Sciences, Yasuj 7591741417, Iran; ^4^Basic Sciences in Infectious Diseases Research Center and Department of Medical Mycology and Parasitology, School of Medicine, Shiraz University of Medical Sciences, Shiraz 7134845794, Iran; ^5^Department of Pathology, Shiraz University of Medical Sciences, Shiraz 7134845794, Iran

## Abstract

*Purpose*. Evaluating the therapeutic effect of topical and intrastromal voriconazole and topical natamycin on* Fusarium* keratitis.* Methods*. 24 rabbits were selected. The stroma of their corneas was inoculated with suspension of* Fusarium solani* species complex. Seven days after injection they were divided into 4 groups randomly: the first group was treated with topical voriconazole (TV) 1% for one week, the second one with one-time intrastromal injection of voriconazole (ISV) 50 microgram/0.1 mL, and the third group with topical gel of natamycin (TN) for one week, and the last one did not receive any antifungal treatment. Finally the eyes were enucleated and sclerocorneal buttons were sent for histological and microbiological examinations.* Results*. After treatment the ISV group and TN group showed significantly lower clinical score and colony forming units than the control group (*P* = 0.040 and *P* = 0.026, resp.), but there was statistically no significant difference between control and TV groups (*P* = 0.249) or between ISV and TN groups (*P* = 0.665). In pathological evaluation, fewer chronic inflammations were reported in 2 of the 3 buttons from TV group and 3 of the 3 buttons from ISV and TN groups in comparison with the control group.* Conclusion*. Intrastromal injection of voriconazole seems to be effective in treatment of* Fusarium* keratitis as much as topical natamycin and these are more effective than topical voriconazole.

## 1. Introduction

Infectious keratitis is one of the important causes of corneal blindness which is a major public health problem worldwide [1]. The incidence of fungal keratitis varies around the world and is more prevalent in areas with hot humid climates. Many species of fungi exist as part of the normal ocular surface microbiota [2]. However, under some circumstances such as corticosteroid therapy and trauma to the eyeball, these fungi might invade the eye and cause fungal ocular infections [1].

Among these fungi,* Fusarium* species are the most frequent cause of fungal keratitis and account for up to one-third of these infections. They are fast-growing hyalohyphomycetes that have been isolated from soil, plants, and water [3]. Most incidences of* Fusarium* keratitis are caused by an eye injury with vegetative matter, such as trauma to the eye with a palm branch [4].* Fusarium* keratitis infections through contact lens wear have been reported, but they are less prevalent [5].

*Fusarium solani* species complex (FSSC) can cause severe types of fungal keratitis because of its high level of virulence and its resistance to antifungal medications [6]. Keratitis caused by FSSC may lead to serious complications such as endophthalmitis, descemetocele, perforation, and blindness [7].

Considering its difficult diagnosis, fungal keratitis is one of the most serious and hazardous forms of corneal infections. Moreover, antifungal drugs are not as forceful and effective as antibacterial agents. Antifungal drugs have little corneal penetration and low efficiency [8].

Different classes of antifungals including polyenes, triazoles, and echinocandins have been used previously in the treatment of fungal keratitis [9]. In India, natamycin is used frequently as the mainstay of treatment [10]. Second-generation triazoles such as posaconazole and voriconazole seem to be effective in the treatment of ocular or corneal fungal infections [11].

Few studies are available about the efficacy of these drugs on fungal keratitis and the route of prescription. One of the recently suggested routes of prescription is intrastromal injection of voriconazole. Siatiri et al. [12] reported results of intrastromal voriconazole injection in the treatment of two patients with recalcitrant* Fusarium* keratitis. They concluded that intrastromal injection of voriconazole together with topical voriconazole effectively reduced the infiltration size and controlled the infection in patients with* Fusarium* keratitis.

In this study, we aimed to evaluate the therapeutic effect of intrastromal voriconazole on* Fusarium* keratitis and compare it with topical voriconazole and topical natamycin.

## 2. Materials and Methods

This study was performed at the Department of Ophthalmology at Khalili Eye Hospital (Shiraz University of Medical Sciences, Shiraz, Iran) and was approved by ethics committee of Shiraz University of Medical Sciences.

FSSC was isolated from a patient with fungal keratitis. The isolate was subcultured on potato dextrose agar (Merck, Germany) plates at 28°C to induce sporulation. The conidia were harvested by washing the surface of the colonies with 0.1% Tween 80 in the sterile physiological saline and filtering the suspension through 2 layers of sterile gauze to remove hyphal residue. The spore suspensions were then transferred to Eppendorf tubes and centrifuged at 10000 ×g to pellet the conidia. The supernatant was carefully removed, and the conidia were resuspended with sterile normal saline to yield the final inoculum of 8.6 × 10^4^ colony forming units (CFU) per milliliter. The number of conidia was measured by hemocytometer and their viabilities were determined and confirmed by quantitative plating of serial dilutions of stock inoculum.

Twenty-four New Zealand white rabbits weighing 2.5 to 3 kg were selected from the animal laboratory of Shiraz University of Medical Sciences. The study was restricted to rabbits that did not have any kind of keratitis. Rabbits with any kind of corneal opacity were excluded from our study. They were systemically anesthetized with 50 mg/kg of intramuscular ketamine hydrochloride and 5 mg/kg of xylazine before all interventions. The stromas of their right corneas were inoculated with 0.1 mL suspension of FSSC (10^3^ CFU/mL under sterile condition in the animal operating room).

**Figure 1:**
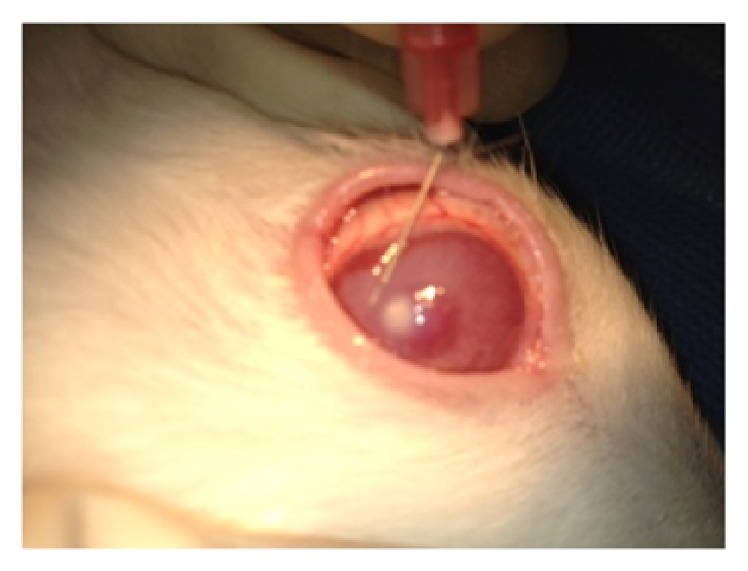
Intrastromal injection of voriconazole 50 microgram/0.1 mL in the operating room.

One week after injection they were checked for fungal keratitis and the size of corneal ulcer and corneal clouding were measured. The rabbits were randomly divided into four groups: the first group received topical voriconazole 1% once each hour (Q1h) (except from 1 a.m. to 7 a.m.) for one week, the second one received a single injection of intrastromal voriconazole 50 microgram/0.1 mL (Figure 1), the third group received topical gel of natamycin Q1h (except from 1 a.m. to 7 a.m.) for one week, and the last group did not receive any antifungal treatment and was considered as the control group. All groups received chloramphenicol drops four times a day for 2 weeks for prophylaxis of bacterial superinfection.

The eyes in four groups were examined at day 7 (baseline) and at day 14 (7 days after beginning of treatment, before enucleation) using an operative biomicroscope.

The extent of keratitis was evaluated by a masked observer. The severity of keratomycosis in the rabbits was scored with the scoring system described by Wu et al. [13]. Each of the following criteria was graded: area of corneal opacity, density of opacity, and surface regularity (Table 1). A normal cornea was given a score of 0 in each category and a summation score of 0. The scores from these categories were tallied for each cornea to show a possible total score ranging from 0 to 12. A total score of 5 or less was considered mild, a total score of 6 to 9 was categorized as moderate, and a total score of more than 9 was considered severe.

On day 14 the eyes were enucleated and sclerocorneal buttons were sent for microbiological and histological examinations. In each of the examined groups, the corneal buttons were weighed, homogenized, and inoculated onto the Sabouraud dextrose agar plates. Following 2–7 days of incubation at 28°C, CFUs/g were determined and compared.

The specimens were immediately inoculated onto the Sabouraud dextrose agar plates and incubated at 28°C for 7 days. The quantity of colonies in each group was determined and compared.

The corneal buttons were fixed in 10% buffered formalin for 24 hours. Then, the buttons were halved and underwent routine tissue processing and tissue block preparation. 5 *μ*m thick tissue sections were prepared and stained with hematoxylin and eosin.

The sections were examined under light microscope (Olympus BX41). Presence or absence of ulceration, fungal hyphae, type and severity of inflammation, extent of stromal scarring and vascularization, and characteristics of infiltrating cells were evaluated.

The data were presented as mean ± SD. The statistical analyses of differences between different groups were performed using the Tukey HSD and Mann-Whitney tests. The Wilcoxon test was used to compare the results for each group between days 7 and 14. *P* < 0.05 was considered as significant. All statistical analyses were done using SPSS software, version 12.

## 3. Results

*Fusarium* corneal ulcer was developed in all eyes one week after intrastromal injection of fungal suspension. Tables 2 and 3 show the mean clinical scores obtained from each group on days 7 and 14.

Kruskal-Wallis one-way analysis of variance showed that statistically there was no significant difference between these four groups at baseline. Mann-Whitney *U* test showed that, on day 14, the difference between the control group and intrastromal voriconazole group was significant statistically. The difference between control group and natamycin group was also significant, but there was statistically no significant difference between the control and the topical voriconazole group or between the intrastromal voriconazole and natamycin groups (Table 4). These data indicate that rabbits in the intrastromal voriconazole and natamycin groups developed infection with less severity than the control and topical voriconazole groups.

The mean ± SD weight of the samples sent to the microbiological laboratory was 0.1494 gr ± 0.0085. The values about the colony forming units (CFUs) that were obtained from the cultured samples are given in Table 5.

Tukey's HSD test showed a statistically significant difference between the control and intrastromal voriconazole groups (Table 6). There was a significant difference between the control and natamycin groups, but the differences between the control and topical voriconazole and between intrastromal voriconazole and natamycin groups were not significant statistically. These data indicate that* Fusarium* proliferation was significantly lower in intrastromal voriconazole and natamycin groups.

In pathological evaluation of the control group similar findings including moderate chronic inflammation, moderate eosinophils, and anterior stromal vascularization were found. The pathological comparison of the experimental groups with the control group is shown in Table 7. Plasma cells, lymphocytes, neutrophils, eosinophils, and mast cells were the inflammatory cells that were investigated.

In pathological evaluation, fewer chronic inflammations were reported in 2 of the 3 buttons from the topical voriconazole group and 3 of the 3 buttons from the intrastromal voriconazole and natamycin groups in comparison with the control group. In 1 of the 3 buttons from the topical voriconazole group, 2 of the 3 buttons from the intrastromal voriconazole group, and 3 of the 3 ones from the natamycin group, fewer eosinophils and anterior stromal vascularization were reported.

## 4. Discussion

Fungal keratitis is an important cause of ocular morbidity and blindness. The diagnosis of these infections is very difficult and currently the therapy for fungal diseases is not as forceful and effective as antibacterials. Antifungal drugs have little corneal penetration and low efficiency [7]. Alexandrakis et al. [14] reported that* Fusarium* spp. were the most common isolates in progressive keratitis. Lin et al. [15] reported that almost 70% of patients with deep lesions of* Fusarium* keratitis do not respond to medical therapy alone. FSSC is very virulent and can destroy an eye completely within a few weeks because the infection is usually severe and perforation, deep extension, and malignant glaucoma may supervene. The most commonly used topical medications for* Fusarium* keratitis are azole derivatives and natamycin.

Prajna et al. [16] found no difference in three-month best spectacle corrected visual acuity or scar size between natamycin- and voriconazole-treated patients in* Fusarium* keratitis. However voriconazole-treated patients were more likely to perforate than natamycin-treated cases. Prajna et al. [17] stated that natamycin treatment was associated with significantly better clinical and microbiological outcomes than voriconazole treatment for smear-positive filamentous fungal keratitis, especially in patients with* Fusarium* infection. In this study we found that topical natamycin-treated* Fusarium* ulcers had fewer clinical scores, CFU, and less severe pathological findings than topical voriconazole-treated patients.

Other researchers reported that all of* Fusarium* species were sensitive to natamycin, but that did not translate to good clinical outcome in patients with* Fusarium* keratitis irrespective of early or late presentation. This probably indicates the poor penetration of natamycin especially in the presence of advanced fungal keratitis affecting deeper layers of the cornea [10]. Several studies have evaluated the effect of more potent drugs and delivery to the site of action in the posterior stroma using intrastromal or intracameral injections [18, 19].

Siatiri et al. [12] described the outcome in 3 patients with recalcitrant* Fusarium* keratitis and reported that intrastromal injection of voriconazole together with topical voriconazole is effective in reducing the infiltration size and control of the infection. Sharma et al. [20] offered intrastromal injection of voriconazole as a modality of treatment for managing cases of recalcitrant fungal keratitis. In this study we found that, in rabbits with* Fusarium* keratitis, intrastromal injection of voriconazole seems to be as effective as topical natamycin. It had lower clinical score, CFUs and fewer chronic inflammations, eosinophils, and anterior stromal vascularization than topical voriconazole. Since the cost of intrastromal injection of voriconazole is less than the frequent use of topical natamycin, it appears to be more economical to inject intrastromal voriconazole in* Fusarium* corneal ulcers. On the other hand, the use of one-time intrastromal injection of voriconazole is more comfortable than applying topical natamycin every hour. Moreover, intrastromal injection of voriconazole yields higher patient compliance. We believe that intrastromal voriconazole can be useful in the treatment of* Fusarium* keratitis especially in ulcers that do not respond to other treatment modalities. We propose further studies on different concentrations of intrastromal voriconazole to investigate their influence on* Fusarium* keratitis to find the most effective concentration.

## Figures and Tables

**Table 1:** Quantitative scoring system for rabbit *Fusarium* keratitis.

	Grade I	Grade II	Grade III	Grade IV
Area of corneal opacity	1%–25%	26%–50%	51%–75%	76%–100%
Density of corneal opacity	Slight cloudiness, outline of iris and pupil being discernable	Cloudy, but outline of iris and pupil remains visible	Cloudy, opacity not uniform	Uniform opacity
Surface regularity	Slight surface irregularity	Rough surface, some swelling	Significant swelling, crater or serious descemetocele formation	Perforation or descemetocele

**Table 2:** Mean clinical scores obtained from each group on day 7.

Group	Factor	*N*	Minimum	Maximum	Mean	SD
C	Clinical score on day 7	6	6.00	8.00	7.1667	0.75277
TV		6	6.00	8.00	7.1667	0.98319
ISV		6	6.00	8.00	7.1667	0.75277
N		6	6.00	8.00	7.0000	0.89443

C: control group, TV: topical voriconazole, ISV: intrastromal voriconazole, N: natamycin, SD: standard deviation.

**Table 3:** Mean clinical scores obtained from each group on day 14.

Group	Factor	*N*	Minimum	Maximum	Mean	Std. deviation
C	Clinical score on day 14	6	4.00	10.00	6.8333	2.22860
TV		6	4.00	10.00	5.5000	2.25832
ISV		6	3.00	5.00	4.3333	0.81650
N		6	3.00	5.00	4.1667	0.75277

C: control group, TV: topical voriconazole, ISV: intrastromal voriconazole, N: natamycin.

**Table 4:** Comparisons between the four groups in terms of clinical score on day 14.

Groups	Mann-Whitney *U*	Significance
C-TV	11.000	0.249
C-ISV	5.5	0.040
C-N	4.5	0.026
TV-ISV	12.5	0.337
TV-N	10	0.167
ISV-N	15.5	0.665

C: control group, TV: topical voriconazole, ISV: intrastromal voriconazole, N: natamycin.

**Table 5:** The values of the colony forming units obtained from the cultured samples.

Group	Factor	*N*	Minimum	Maximum	Mean	SD
C	Colony forming unit/g	6	48.0	52.0	50.333	1.8619
TV		6	0.00	51.0	24.667	27.0382
ISV		6	0.00	48.0	8.000	19.5959
N		6	0.00	0.00	0.0000	0.00000

C: control group, TV: topical voriconazole, ISV: intrastromal voriconazole, N: natamycin, SD: standard deviation.

**Table 6:** Tukey's HSD test analysis of colony forming units.

Group I	Group J	Mean difference (I − J)	SE	Significance	95% confidence interval	
					Lower bound	Upper bound
Control	ISV	42.3333	9.6546	0.002	15.311	69.356
	N	50.3333	9.6546	0.000	23.311	77.356

ISV: intrastromal voriconazole, N: natamycin, SE: standard error.

**Table 7:** The pathological comparison of 9 corneal buttons of the experimental groups with the control group (3 corneal buttons from each experimental group).

	TV	ISV	N
Chronic inflammation	Similar to C	Fewer than C	Fewer than C
Eosinophils	Similar to C	Fewer than C	Fewer than C
Anterior stromal vascularization	Similar to C	Fewer than C	Fewer than C
Chronic inflammation	Fewer than C	Fewer than C	Fewer than C
Eosinophils	Fewer than C	Fewer than C	Fewer than C
Anterior stromal vascularization	Fewer than C	Fewer than C	Fewer than C
Chronic inflammation	Fewer than C	Fewer than C	Fewer than C
Eosinophils	Similar to C	Similar to C	Fewer than C
Anterior stromal vascularization	Similar to C	Similar to C	Fewer than C

C: control group, TV: topical voriconazole, ISV: intrastromal voriconazole, N: natamycin.
